# Non-invasive characterization of complex coronary lesions

**DOI:** 10.1038/s41598-021-86360-6

**Published:** 2021-04-14

**Authors:** Madhurima Vardhan, John Gounley, S. James Chen, Eric C. Chi, Andrew M. Kahn, Jane A. Leopold, Amanda Randles

**Affiliations:** 1 Department of Biomedical Engineering, Duke University, Durham, NC 27705 USA; 2 Computational Sciences and Engineering Division, Oak Ridge National Laboratory, Oak Ridge, TN 37830 USA; 3 Department of Medicine/Cardiology, University of Colorado AMC, Aurora, CO 80045 USA; 4 Department of Statistics, North Carolina State University, Raleigh, 27695 USA; 5 Division of Cardiovascular Medicine, University of California San Diego, San Diego, 92103 USA; 6 Division of Cardiovascular Medicine, Brigham and Women’s Hospital, Harvard Medical School, Boston, MA 02115 USA

**Keywords:** Interventional cardiology, Computational science, Fluid dynamics, Computational models

## Abstract

Conventional invasive diagnostic imaging techniques do not adequately resolve complex Type B and C coronary lesions, which present unique challenges, require personalized treatment and result in worsened patient outcomes. These lesions are often excluded from large-scale non-invasive clinical trials and there does not exist a validated approach to characterize hemodynamic quantities and guide percutaneous intervention for such lesions. This work identifies key biomarkers that differentiate complex Type B and C lesions from simple Type A lesions by introducing and validating a coronary angiography-based computational fluid dynamic (CFD-CA) framework for intracoronary assessment in complex lesions at ultrahigh resolution. Among 14 patients selected in this study, 7 patients with Type B and C lesions were included in the complex lesion group including ostial, bifurcation, serial lesions and lesion where flow was supplied by collateral bed. Simple lesion group included 7 patients with lesions that were discrete, $<10\hbox {mm}$ long and readily accessible. Intracoronary assessment was performed using CFD-CA framework and validated by comparing to clinically measured pressure-based index, such as FFR. Local pressure, endothelial shear stress (ESS) and velocity profiles were derived for all patients. We validates the accuracy of our CFD-CA framework and report excellent agreement with invasive measurements ($n=14, R^2 = 0.6, p = 0.0013$). Ultra-high resolution achieved by the model enable physiological assessment in complex lesions and quantify hemodynamic metrics in all vessels up to 1mm in diameter. Importantly, we demonstrate that in contrast to traditional pressure-based metrics, there is a significant difference in the intracoronary hemodynamic forces, such as ESS, in complex lesions compared to simple lesions at both resting and hyperemic physiological states [n = 14, $p=0.03$]. Higher ESS was observed in the complex lesion group ($7.0\pm 4.7$ Pa) than in simple lesion group ($4.8\pm 3.6$ Pa). Complex coronary lesions have higher ESS compared to simple lesions, such differential hemodynamic evaluation can provide much the needed insight into the increase in adverse outcomes for such patients and has incremental prognostic value over traditional pressure-based indices, such as FFR.

## Introduction

Complex coronary lesions, such as bifurcation and ostial lesions, are found in more than 20% of patients suffering from coronary diseases who are at high risk of developing secondary major adverse cardiac events^1–4^. Owing to their anatomic, physiological and functional difficulties, conventional diagnostic metrics are inadequate for assessing disease progression in complex lesions^1–4^. Secondary hemodynamic biomarkers are promising metrics for predicting outcome of complex lesions and thus guiding future treatment decisions. Thus, there is an urgent need to determine intracoronary hemodynamic quantities that can differentiate complex coronary lesions on a per-patient basis. Non-invasive computational diagnostic approaches can be particularly useful in assessing complex lesions. However, despite their usefulness in simple, single coronary lesions, the utility of these methods to diagnose complex coronary lesions remains elusive as these patients have been excluded in most clinical trials using such computational models^5,6^. In this study, we present a computational fluid dynamic (CFD) framework that accurately determines coronary arterial tree physiology at ultra-high resolutions. This method enables quantitative evaluation of intra-arterial hemodynamics in complex coronary diseases, such as ostial lesion, bifurcation lesion and serial lesion, with high accuracy and prediction reliability for the first time. The accuracy of our framework is determined by comparing derived diagnostic metrics to clinical measurements, such as fractional flow reserve (FFR) in both simple and complex coronary lesions.

While it is known that using conventional diagnostic metrics to guide revascularization planning can reduce the need for unnecessary intervention in single main branch lesions, such benefits for complex coronary lesions lack compelling clinical evidence. Further, complications such as in-stent restenosis (ISR) occur in 30% of the bifurcation lesion cases following intervention^1,3,4^. A similar story prevails for ostial lesions which respond poorly to conventional interventional procedures and present higher procedural and medium-term complications^1,3,4,7,8^. Due to the morphological complexity of these lesions, data on the intracoronary hemodynamic characteristics in complex lesions remains unavailable^3,4,7,8^. This study addresses this limitation by proposing a CFD framework that naturally incorporates realistic patient anatomy and derives FFR and the local flow quantities, such as velocity and shear stress. FFR is the trans-stenotic pressure ratio measured to determine whether an arterial blockage should be stented. To supplement FFR, accurate biomechanical models can be used to establish a clinically-relevant metric to diagnose and predict disease progression.

**Figure 1:**
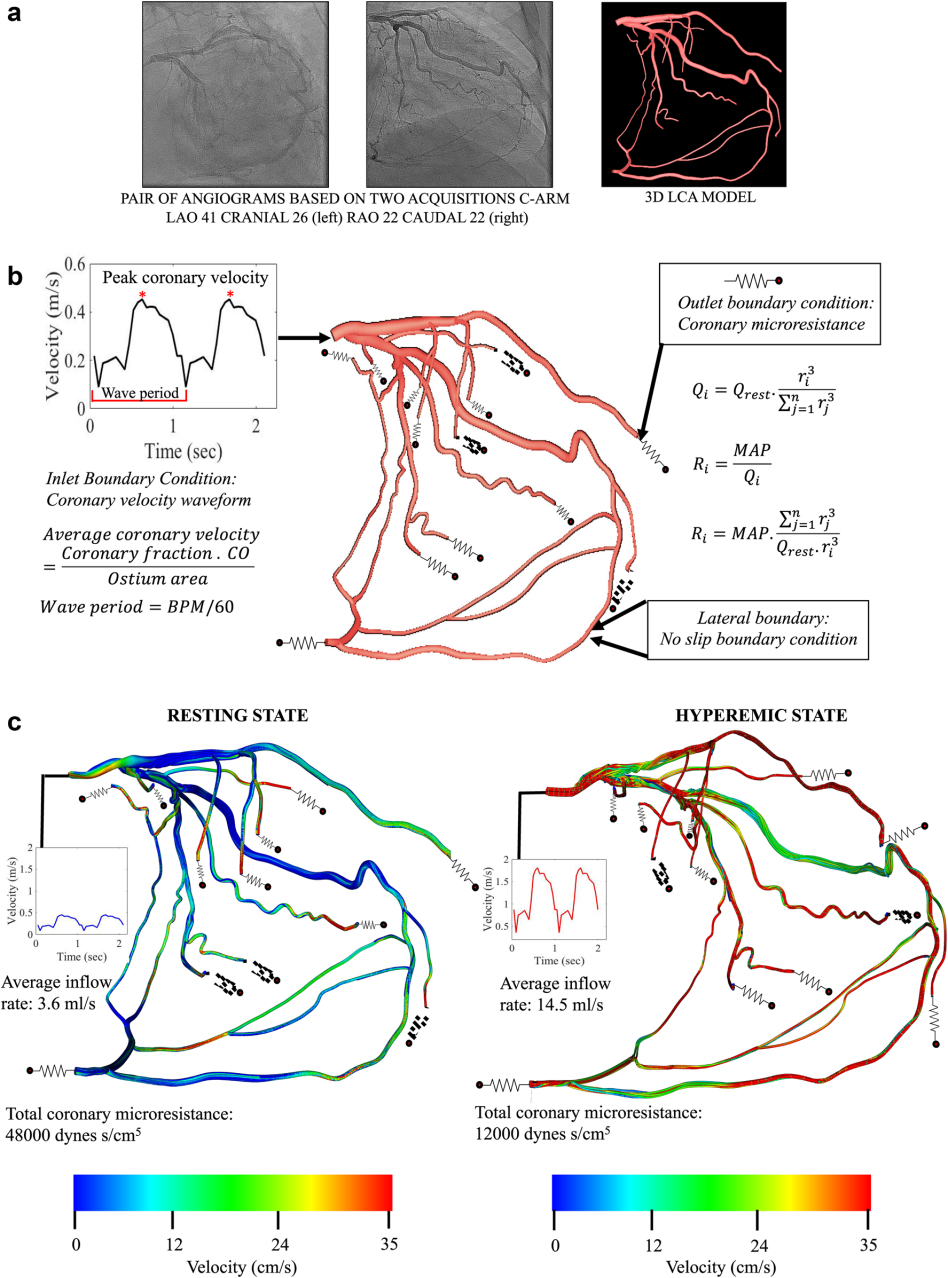
Central illustration Computational fluid dynamic (CFD) methodology applied to biplane coronary angiography (CA) data in a left coronary circulation that supplies RCA vascular bed via collaterals. (**a**) Two biplane CA views at a particular angle for a left coronary arterial tree with collateral flow with the corresponding 3D reconstructed geometry. (**b**) CFD model of blood flow applied to the reconstructed geometry from (**a**). Boundary conditions are applied at the inlet (transient velocity waveform), outlet (lumped parameter for arterial microcirculation) and lateral surfaces (zero velocity or no slip boundary condition). (**c**) Modeling resting and hyperemic coronary circulation with a patient-data tuned velocity waveform and total coronary microresistance, 48000 dynes s/cm^5^ and 12000 dynes s/cm^5^ at resting and hyperemic states, respectively.

Complex lesions are challenging to model with existing computational frameworks due to anatomic and functional difficulties which require (1) a high-resolution imaging modality and (2) physiological parameters that can lead to instabilities and inaccuracies in numerical approaches. Therefore, to fully elucidate the anatomy-function relationship of arterial hemodynamics in complex lesions, our computational framework comprises two steps: first, reconstructing complete arterial trees using coronary angiography (CA) imaging data and, second, performing hemodynamic simulations using HARVEY (v2) - a massively parallel CFD solver. Using 3D geometries derived from CA data with secondary and tertiary bifurcations is a key development and has required integration of a biplane reconstruction algorithm^9^. CA offers high spatial and temporal resolution, superior to CT angiography, and can therefore can be used to incorporate the anatomical detail needed for complex arterial anatomy^10^.

Our CFD framework allows physiological maps to be overlaid on conventional imaging data which can be directly used to determine hemodynamic differences across the entire arterial tree. The size of the smallest vessels that can be modeled imposes restrictions on maximum achievable resolution due to the large computational load^11^. Using parallel computing, this framework enables ultra-high resolution physiological modeling in reasonable execution times, thus meeting the high computational demand needed for these *in silico* experiments. Fast and accurate non-invasive functional arterial assessment is achieved by using the massively parallel CFD solver HARVEY (v2)^12,13^. With this CFD-CA framework, we expect to non-invasively derive diagnostic metrics, such as FFR with lower errors and reclassification rates, and resolve local hemodynamics for pathophysiological analysis in all identifiable vessels in an angiogram. We believe such hemodynamic profiling at the clinical level will be especially useful in complex coronary lesion which continue to suffer from high rate of surgical complications and major adverse cardiac events^1,3,4,7,8^.

**Table 1:** Validating CFD-CA derived FFR by comparing to invasively measured FFR.

Lesion	Vessel	Clinical resting gradient	CFD-CA resting gradient	Absolute percent error	Invasive FFR	CFD-CA FFR	Absolute percent error	0.8 cutoff agreement
**Simple lesions**								
Type A Minimal	LAD	0.93	0.94	0.6	0.83	0.83	0.0	Agree
Type A Intermediate	RCA	0.97	0.98	1.0	0.87	0.95	9.2	Agree
Type A Intermediate	LCx	0.96	0.97	1.0	0.89	0.89	0.0	Agree
Type A Mild	LAD	0.95	0.95	0.0	0.88	0.90	2.3	Agree
Type A Minimal	RCA	0.91	0.90	0.7	0.81	0.80	1.2	Agree
Type A Severe	RCA	0.98	0.92	6.0	0.9	0.81	10.0	Agree
Type A Intermediate	RCA	1	0.95	5.0	0.95	0.89	6.3	Agree
**Complex lesions**								
Ostial and proximal serial lesions	RCA	0.88	0.88	0.0	0.72	0.69	4.2	Agree
Serial lesion in LAD	LAD	0.96	0.99	3.1	0.89	0.96	7.9	Agree
Supplies RCA vascular bed via collaterals	LCx	1	0.98	2.0	0.95	0.98	3.2	Agree
Serial lesions	LAD	0.82	0.72	12.2	0.58	0.66	13.8	Agree
Bifurcation lesion involving D1	Left main	0.92	0.95	3.3	0.84	0.91	8.3	Agree
Serial lesions in RCA	RCA	0.98	0.93	5.1	0.82	0.70	14.6	Disagree
Serial lesions LAD	LAD	0.93	0.97	4.3	0.83	0.94	13.3	Agree
				3.16 ± 0.03%			6.73 ± 0.05%	

Left anterior descending: LAD, left circumflex: LCx, right coronary artery: RCA, diagonal 1: D1. Minimal stenoses: 1–24%, mild stenoses: 25–49%, intermediate stenoses: 50–69% and severe stenoses: 71–99%.

**Figure 2:**
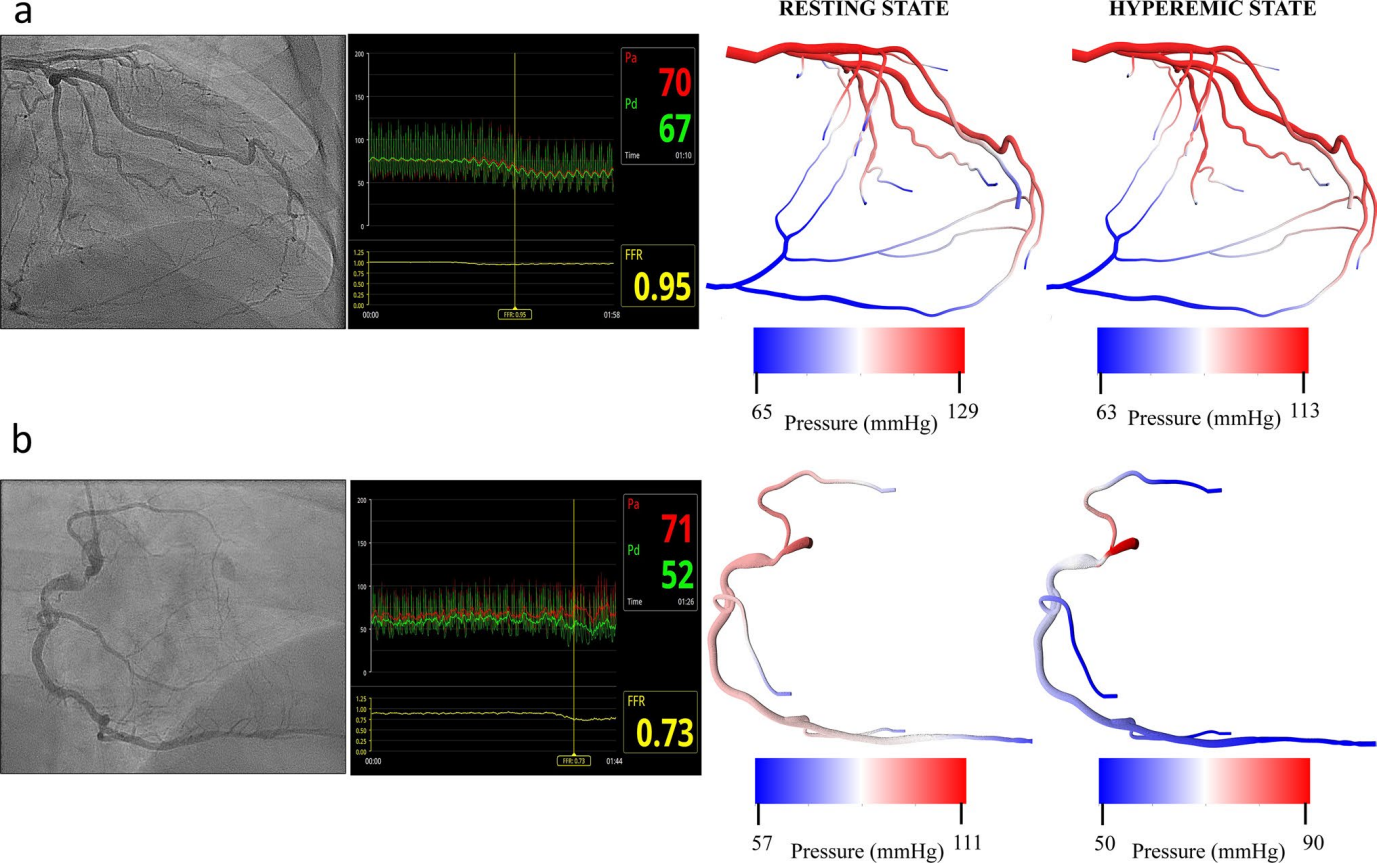
Pressure gradient in arterial geometries at resting and hyperemic conditions. (**a**) Left coronary artery with a non-significant lesion (<50%) and collateral vessels. Pressure gradient at rest and hyperemic states derived from CFD-CA methodology does not change significantly, as shown on the right. (**b**) Right coronary artery with a significant proximal lesion (>70%). Pressure gradient at rest and hyperemic states derived from CFD-CA methodology changes significantly, as noted on the right.

## Results

### Accurate computational modeling of fluid dynamics using conventional angiogram data

This study uses 14 patient datasets acquired from Brigham and Women’s Hospital which had 2D angiograms and invasively measured FFR values at the time of coronary angiography (Table 1). Among these, 7 cases are complex coronary lesion cases including ostial, left main, bifurcation, serial lesions and target lesion vessels supplied by collateral flow. The remaining 7 cases are Type A coronary lesions, which are discrete, <10mm long and readily accessible with varying degree of percent stenoses in the left circumflex, left anterior descending or right coronary arteries. The 3D coronary artery geometry, as depicted in Fig. 1a, was reconstructed from patient angiograms using our 3D biplane reconstruction algorithm^9,14,15^. To validate the reconstructed geometries, an experienced interventional cardiologist compared the anatomy of the reconstructed arterial models to the original patient angiogram used as input to the reconstruction algorithm. The arterial architecture was compared in terms of number of vessels, vessel type (main vessels, side branches and collateral vessels), branching patterns (bifurcation and trifurcation) and stenoses anatomy (location, length and severity). A quantitative comparison was established by aligning the reconstructed models to match arterial orientation in the angiograms and measuring the ostium diameter and minimal luminal diameter of the lesion using ImageJ (v1.52k)^16^. The numerical results of these measurements can be found in Supplementary Table 1. Subsequently, upon satisfactory anatomic agreement between reconstructed arterial models and patient angiograms, the 14 models were used as inputs for HARVEY (v2) simulations. We developed our framework for deriving 3D hemodynamic variables from 2D angiograms using our massively parallel hemodynamic solver, HARVEY (v2). This simulator relies on the implementation of the lattice Boltzmann method (LBM), an alternative solver of the Navier-Stokes equations^12^. LBM has been extensively used to efficiently model hemodynamics in patient-specific geometries^17–22^. More recent studies have leveraged LBM-based CFD solvers to derive pressure fields required to compute FFR^23,24^. In contrast to other CFD solvers, LBM-based solvers allow efficient distribution of computational load and high-resolution simulations on millions of processors^13,17^. HARVEY (v2) used the 3D coronary arterial mesh in standard stereolithography (STL) format as input to the model.

**Figure 3:**
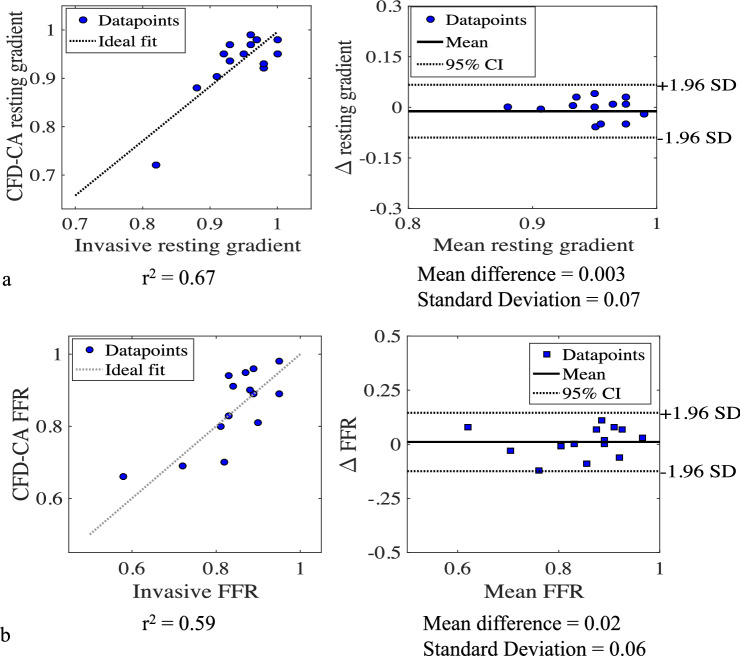
(**a**) Correlation between invasive resting gradient and CFD-CA resting gradient (left) and corresponding different plot determined from Bland Altman analysis (right). (**b**) Correlation between invasive FFR and CFD-CA FFR (left) and corresponding different plot determined from Bland Altman analysis (right).

The blood vessels were modeled as rigid walls with a no-slip boundary condition on the flow^25,26^. The inlet boundary condition was applied at the coronary ostium where we imposed a volumetric flow waveform with a Poiseuille profile to attain transient inflow. Patient-derived velocity waveforms were constructed using heart rate, cardiac output (CO) and coronary dominance measures for each patient under resting or basal flow conditions (Fig. 1b). At the coronary outlets, a lumped parameter model was prescribed using microcirculation resistance. The coronary arterial microcirculation resistance for all patient-specific arterial geometries was computed based on the vessel diameter, mean coronary flow rate and mean aortic pressure retrieved from patient records by enforcing the allometric scaling laws and the relationship between pressure and flow^25^. Specifically, unique resistance values were computed for each coronary outlet by applying an equivalent resistor model that obeys the structure-function relationship (Fig. 1b)^27^. As per the resistor model, under resting flow conditions, blood vessel resistance is a function of the vessel size and viscosity, which was kept constant during the course of the simulation (Fig. 1c). Resistance is thus computed as the ratio between mean arterial pressure and volume fraction. Volume fraction of each outlet is evaluated based on the vessel size and total coronary volume. Using equations 4 and 5, we note that the resistance of each outlet is inversely related to the vessel size, that is $R \propto 1/d^4$, which is also consistent with the morphometry laws^25,28–30^. The final step is modeling the hyperemic state, which is induced by the pharmacological effects of vasodilators such as adenosine. During hyperemia, two metrics critically change due to vessel dilation - resistance and velocity (Fig. 1c)^31^. Total coronary resistance is 0.25 times of the basal resistance; consequently, peak velocity is 3.5 - 4.2 of basal peak velocity, with intravenous administration of 140 $\mu$g/kg/min adenosine, the dose used for FFR measurement^25,31^. A summary of parameters simulated for each patient is shown in Supplementary Table 2.

**Figure 4:**
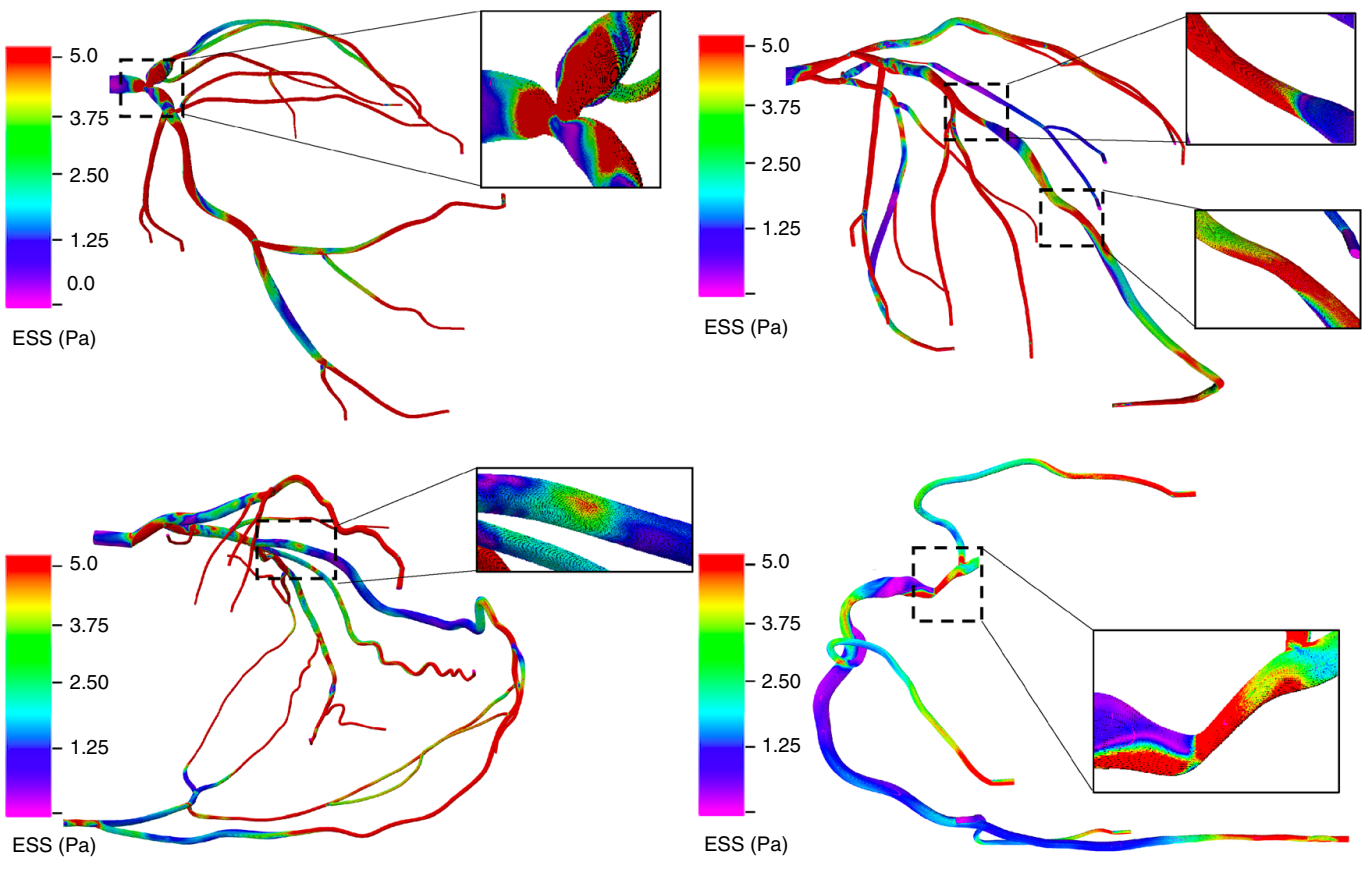
Endothelial shear stress in arterial geometries with different complex coronary lesions: (top left) bifurcation lesion (top right) serial lesion (bottom left) RCA vascular bed via collaterals (bottom right) ostial lesions. The inset marks the region of the stenosis.

As a first evaluation, we use these simulation parameters to perform simulations at resting and hyperemic states for all cases. At resting state, we note that in each patient case, pressure decreases along the length of the arterial tree towards the most distal segments. In particular, for non-atherosclerotic segments and vessels with lesion severity $<50\%$, a slight gradual decrease in pressure is noted (Fig. 2a). However, relative to post-stenotic region, the pressure is elevated in pre-stenotic regions for cases with significant stenoses severity ($>70\%$) (Fig. 2b) due to the funnel-shaped anatomy. This result can be explained with Bernoulli’s law as higher velocity in the region of stenoses is associated with a decrease in pressure and is consistent with findings reported by other studies^32^. This overall trend remains consistent at the hyperemic state for vessels with lesion severity $<50\%$; however, a much greater pressure gradient is noted for cases with significant stenoses severity $>70\%$ (Fig. 2b). To evaluate the extensibility of our resting and hyperemic simulation setup, we applied it to complex coronary stenoses, such as ostial lesions, serial lesions, and bifurcation lesions (Supplementary Table 2). The pressure gradients in the diseased vessel were noted to follow similar trends strongly influenced by the severity of the lesion. Consequently, these results demonstrate that our computational modeling of fluid dynamics using conventional angiogram data is both feasible and extensible to complex coronary lesion types and collateral flow. It provides quantitative predictions of pressure gradients that are in accordance with invasively measured pressure gradients (Fig. 2). Therefore, our next step was to validate the CFD-CA FFR measurement by comparing it with invasive FFR values in all 14 patient datasets.

**Figure 5:**
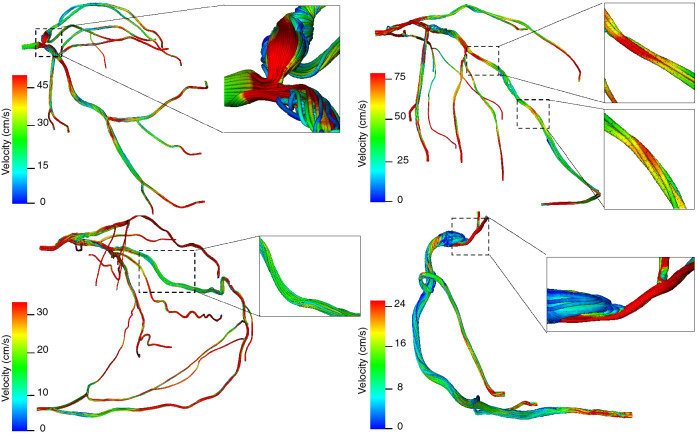
Velocity streamlines in arterial geometries with different complex coronary lesions: (top left) bifurcation lesion (top right) serial lesion (bottom left) RCA vascular bed via collaterals (bottom right) ostial lesions. The inset marks the region of the stenosis.

### Quantitative validation of CFD-CA predictions for FFR in complex and non-complex coronary lesions

By using coronary angiogram data and ultra-high resolution of CFD simulations, we were able to account for the anatomic and physiological difficulties encountered in modeling complex coronary lesions. The key to the interpretation of our CFD-CA pressure gradient results was our ability to compare them directly with equivalent clinical measurements. Thus, to provide the ground truth data for comparison, we computed the percentage error of the CFD-CA resting gradient and CFD-CA FFR values by directly comparing them to invasive resting gradient and invasive FFR measures, respectively for both complex lesion and Type A lesion groups (Table 1). In complex and non-complex, Type A coronary lesions, the average error for resting gradient was 2.78% and 3.7% respectively, and for FFR the average error was 7.22% and 6.24% respectively. For all cases, the average percent error, median percent error, standard deviation and inter-quartile range is 2.55%, 3.16%, 0.03 and 0.04 for resting gradient and 6.73%, 7.10%, 0.05 and 0.07 for FFR.

To assess whether differences between CFD-CA and invasive measures were statistically significant, we performed Deming regression between CFD-CA resting gradient measurements and invasive resting gradient. Deming regression permits error and variation in both sets of measurements being compared and consequently puts the CFD-CA and invasive measurements on the same footing by not treating either as a gold standard reference. This is in contrast with simple linear regression which assumes the independent variable is measured without error (gold standard) and the dependent variable is measured with error. For the resting gradient data we computed an intercept of $-0.105$ with a 95% confidence interval (CI) of ($-1.286$, 1.075) and a slope of 1.138 with a 95% CI of ($-0.218$, 2.495). Since the 95% CIs contain 0 and 1 respectively for the fitted intercept and slope, we conclude that there were neither statistically significant systematic nor proportional differences between the invasive and CFD-CA measurements. Performing Deming regression between CFD-CA FFR and invasive FFR resulted in a fitted intercept of 0.316 with a 95% CI ($-0.643$, 1.275) and slope of 0.673 with a 95% CI of (-0.338, 1.683). Again since the 95% CIs contained 0 and 1 respectively for the intercept and slope, we conclude that there were neither statistically significant systematic nor proportional differences between the invasive and CFD-CA measurements.

Bland Altman analysis further confirmed the agreement between CFD-CA and invasive measures. Figure 3 shows the correlation between CFD-CA and invasively measured values at baseline (resting gradient) and hyperemic (FFR) states with the corresponding Bland Altman plots. The estimated mean difference is 0.003 for resting gradient and 0.02 for FFR; this minor deviation suggests minimal overestimation and underestimation of invasive resting gradient and invasive FFR, respectively. The 95% CI agreement values were $-0.09$ and 0.07 and $-0.12$ and 0.15 for the resting gradient and FFR, respectively. The agreeability, as drawn by the Bland-Altman plot, shows that all values of the resting pressure gradient and FFR lie within the 95% limits of agreement. Therefore, regression analysis and Bland-Altman plots demonstrate that scatter around the ideal fit and mean are stable as the average increases. Finally, using the two-tailed Mann-Whitney statistical test, we determine that there is no statistical difference between the CFD-CA method accuracy for non-complex and complex lesion (p>0.05). We therefore conclude from these experiments that the CFD-CA framework can provide accurate estimates of pressure gradient in complex lesions.

As 0.8 is the clinical FFR cutoff used to decide if revascularization is warranted, lesions were classified into two categories: cases with invasive FFR>0.8, representing non-significant lesions, and cases with FFR$<=$0.8, denoting lesions causing ischemia and requiring percutaneous coronary intervention (PCI)^33^. The computed CFD-CA FFR was also categorized in the same way and assessed for comparison with invasive FFR (Table 1). We report a high level of agreement between CFD-CA FFR and invasive FFR. Diagnostic accuracy of CFD-CA FFR with 95% confidence was evaluated as follows - sensitivity 92.31% (0.63 to 0.99), specificity 100% (0.15 to 1.00), positive predictive value 100%, and negative predictive value 66.67% (0.23 to 0.93). The overall diagnostic accuracy was 93.33%. The coefficient of variation of invasive FFR has been previously reported as 4.8%^34^. Keeping this internal variation in mind, the diagnostic accuracy of 93.33% for our CFD-CA workflow is encouraging, despite the small cohort of patient cases. Furthermore, keeping the input variables the same, two independent operators using our CFD-CA workflow would obtain identical pressure gradients, thus we report no inter-operator variability. With this validation, we believe that our CFD-CA methodology accurately resolves the underlying physiology for left and right coronary circulation. Therefore, we chose to explore differences between local hemodynamic properties of complex vs. non-complex lesions.

**Figure 6:**
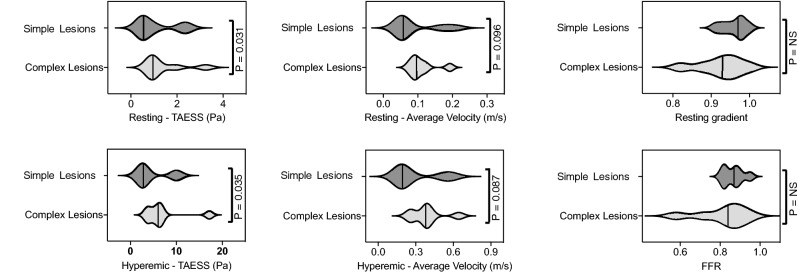
Violin plots to compare time-averaged endothelial shear stress (TAESS), velocity, resting gradient and fractional flow reserve (FFR) in complex versus non-complex lesions at resting and hyperemic physiological states. ESS was averaged over the period of cardiac cycle and is reported as TAESS. Velocity for each patient case was averaged along the length of the stenosis. Solid lack lines identifies the median values.

### Differences in intra-arterial hemodynamics between complex and non-complex coronary lesions

Having validated our CFD-CA workflow, we next tested two hypotheses: (1) local hemodynamics differences exist between complex and non-complex lesions included in this study, and (2) differences in local hemodynamics are exacerbated for complex lesion cases which could help understand worsened outcomes in patients with coronary artery disease.

We test these hypothesis for the specific lesions included in this study. Our rationale for these hypotheses was that previous studies have shown that arterial shear stress,  1.5 Pa, is considered atheroprotective and >2.5 Pa can lead to pro-atherosclerotic plaque rupture^35–37^. Therefore, supplementing the knowledge of FFR with a rich 3D hemodynamic environment, by investigating spatial flow heterogeneity under resting and hyperemic conditions, can help in prospectively evaluating frequently witnessed adverse events related to complex lesions.

We note resting gradient and FFR values between complex and non-complex lesions do not necessarily differ, and local physiological changes that arise directly due to the complicated anatomy of complex lesions could better help understand disease progression (Fig. 6). This phenomenon may occur because resting gradient and FFR are pressure ratios that estimate maximum achievable flow in the vessel, and thus may not provide a complete view of the reduced coronary flow, which is the actual determinant of myocardial ischemia^38^. We tested our hypotheses by analyzing two characteristic hemodynamic risk-factors: endothelial shear stress (ESS) and velocity at resting and hyperemic states^39,40^. ESS was averaged over the period of cardiac cycle and calculated as time-averaged endothelial shear stress (TAESS) and velocity was computed as the circumferential average velocity. Both quantities were then calculated along the entire lesion length. Figures 4 and 5 respectively, display the results for ESS and velocity patterns in different complex lesions: bifurcation, serial, RCA vascular bed supplied through collateral vessel and ostial lesions.

To test our hypotheses, we compared ESS and velocity for patients in complex and non-complex lesion groups. Figure 6 represents violin plots to demonstrate the difference in ESS and velocity for complex vs. non complex lesions under resting and hyperemic conditions. At resting state we observe for complex lesions, ESS and velocity were 1.4 ± 0.9 (Pa) and 0.11 ± 0.04 (m/s) respectively, whereas for non-complex lesions lower magnitude of ESS and velocity were noted, 1.1 ± 0.8 (Pa) and 0.09 ± 0.06 (m/s), respectively. Similar trend was observed at hyperemic flow conditions. For complex lesions ESS and velocity were measured as 7.0 ± 4.7 (Pa) and 0.4 ± 0.1 (m/s) respectively and for non-complex lesions ESS and velocity were found to be 4.8 ± 3.6 (Pa) and 0.3 ± 0.1 (m/s) respectively.

To determine the statistical significance of these hemodynamic differences we used a paired ratio t-test. Pairing between complex and non-complex case was done based on the inlet boundary condition. Specifically, a complex and non-complex case with similar average velocity flow at the ostium was paired. Averaged velocity at the ostium was computed by dividing the cardiac output, determined based on the coronary dominance, and ostium area. The cardiac output, coronary dominance and ostial diameter were measured by an experienced interventional cardiologist. This pairing allowed us to assess the hemodynamic differences arising due to the unique morphology of complex lesions compared to simple single, lesions, despite the similar inlet flow rate. For resting condition, the ratio of complex and non-complex lesions for ESS and velocity was 0.69 ± 0.1 [*p* = 0.03] and 0.73 ± 0.18 [*p* = 0.09] respectively. And at hyperemic conditions, the ratio for ESS and velocity was 0.62 ± 0.2 [*p* = 0.03] and 0.7 ± 0.21 [*p* = 0.08] respectively.

For the specific cases included in this study, the ESS and velocity differences do not arise based just on the stenosis severity, but in fact due to the unique anatomy of complex lesions, since the percentage of stenosis in some complex lesion cases was less severe than the corresponding simple lesion case. In summary we note that for the particular cases in this study, ESS was significantly different between complex and non-complex lesion at both resting and hyperemic conditions and marginal significance was observed for velocity. As high ESS has been shown to predict adverse cardiac events, such as myocardial infarction^41^, we believe these findings can be clinically important and used to guide treatment in patients with complex lesions. However, it is also worth noting that even simple, single lesions with high severity or flow rates could also lead to high ESS and velocity. And as such, these findings could be dependent on the choice of cases. Therefore, to provide a general evidence for ESS and velocity differences between lesion types the range of complex lesion cases should be representative of most anatomic variants and severity of different complex cases, and likewise for simple lesion cases. Overall such an analysis requires a large clinical study.

### Evaluating model robustness of CFD-CA method to variations in clinical measurement

**Figure 7:**
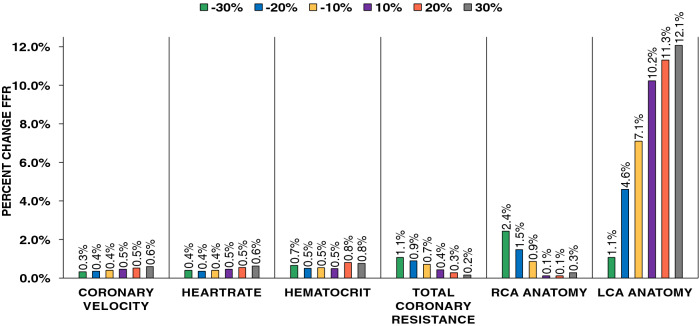
Sensitivity analysis. Coronary anatomy is the driving feature for FFR predictions. Key hemodynamic input parameters: coronary velocity, heartrate, hematocrit and resistance were varied by ±10%, ±20%, and ±30%. To account for geometric influence percent stenosis was varied by ±10%, ±20%, and ±30%.

Personalized computational models require adapting physiological clinical data and patient-derived arterial geometries to input parameters to the mathematical model. In particular for patient-specific hemodynamic simulations presented in this study, 4 key physiological clinical input parameters were used: coronary velocity, heart rate, hematocrit and resistance. To account for how sensitive our CFD-CA predicted FFR values are to variations of each of these parameters for a given patient case, we performed a sensitivity analysis. We expected that depending on the measurement variation in these clinical parameters, our derived FFR values would vary. Thus, we attempted to evaluate how our results respond to any such changes to determine the robustness of our CFD-CA framework. All parameters were individually varied by ±10%, ±20%, and ±30% while keeping the other parameters unchanged. The results of this analysis demonstrate that HARVEY (v2) FFR predictions remained relatively unaltered, <2%, with variations in these clinical parameters for a given patient geometry (Fig. 7). We then evaluated the effect of changes in the arterial anatomy on FFR. We assessed this effect by changing the percentage of stenosis by ±10%, ±20%, and ±30%, and calculating the resulting FFR value for each varied stenosis percentage for an LCA and an RCA geometry. Percent stenosis was used as the feature to study the effect of anatomic change because minimal luminal diameter is a key feature that influences FFR^42^. As anticipated, CFD-CA FFR values were sensitive to anatomic variations attributed to percent stenoses, and FFR variation as high as 12% was noted for the 30% increase in percent stenoses (Fig. 7). Similar sensitivity analysis was performed for ESS and velocity (supplementary Figure 4 and 5, respectively). The results show that ESS and average hyperemic velocity vary with changes LCA/RCA lesion anatomy but compared to FFR these metrics are more sensitive to fluid flow variables, for example cardiac output and hematocrit. Such a trend is expected as cardiac output and hematocrit directly influence inflow velocity magnitude and patterns having a more dramatic influence on ESS and velocity, whereas FFR is not highly sensitive to hemodynamic conditions^34^ These results demonstrate that for a given patient case, coronary anatomy is the driving feature for FFR predictions and small deviations in the clinical parameters have negligible influence making our approach extremely robust to minor variations clinical measurements.

## Discussion

Recent years have witnessed dramatic increase in computational approaches for diagnosing cardiovascular diseases, which continue to dominate healthcare costs and are projected to be over 1 trillion dollars by 2035, according to the American Heart Association. Treatment of complex coronary lesions is involved in more than 20% of the total number of PCI procedures performed and post-interventional complications such as in-stent restenosis occurs in 30% of the cases. PCI challenges for complex lesion are further compounded by the limited accessibility and the high procedural and operational costs of traditional diagnostic methods, which have restricted its wide scale utilization and paved the way for computational methods that can non-invasively derive FFR in routine clinical practice^6,25^. Thus several groups have attempted to apply computational models to medical imaging data to derive FFR with clinical accuracy, but continue to exclude complex lesions^5,6,24,25,43–46^. Furthermore, imaging techniques such as CT angiography, optical coherence tomography and rotational coronary angiography are neither universally available nor routinely performed in clinical practice, which limits the application of computational models that rely on these imaging datasets^24,25,44–46^. This study presents ultra-high resolution personalized CFD simulations to compute conventional diagnostic metrics and intra-arterial physiology in complex and non-complex coronary lesion using widely available coronary angiography imaging data.

### Clinical relevance of ESS in complex coronary lesions

The major findings of this study are: (1) traditional pressure-based metrics, such as FFR, do not significantly differ between non-complex Type A coronary lesions compared to complex lesions, (2) intra-arterial hemodynamics, which were previously unavailable, for complex coronary lesion can be accurately derived from our CFD-CA method, and (3) ESS and intra-lesion velocity are important biomarkers which significantly differ between complex and non-complex lesions and can help understand the reason for such patients developing secondary adverse cardiac events. These findings are critical as high ESS is predictive of clinical outcomes such as myocardial infarction and often alters atherosclerotic plaque characteristics^41,47–49^. This study interrogates ESS as a potential biomarker and builds on the current understanding in literature, while advancing it in a critical way. Capturing coronary physiology described by local hemodynamics throughout the arterial tree is crucial in order to examine the consequences of impaired coronary flow and plaque development. Physiological assessment of the arterial tree can enable an accurate and non-invasive approach in the diagnosis and treatment of coronary artery disease. Patient-specific characterization of local hemodynamic risk factors which have been previously shown to affect formation and progression of atherosclerosis, such as ESS and velocity, can account for complications in certain patient and complex lesion types that otherwise cannot be explained by ubiquitous procedural factors^39,40^. Such characterization is important because local hemodynamic metrics hold the potential to be clinically invaluable to physicians as traditional diagnostic metrics, such as FFR, do not differ between complex lesion anatomies relative to Type A lesions which have simpler morphology. Thus, by providing information about known hemodynamic risk factors, our CFD-CA methodology can aid clinical decision-making. In particular, spatial heterogeneity of local hemodynamic quantities holds enormous clinical relevance as it could provide grounds for prophylactic interventions and potentially avert any future adverse cardiac events, such as myocardial infarction^39,41^.

### Evaluation of intracoronary hemodynamics using CFD-CA framework

The capability of our method to assess complex lesions comes from combining high-resolution coronary angiographic imaging data and a massively parallel hemodynamic simulator. CA has higher spatial and temporal resolution compared to other commonly used imaging modalities in 3D coronary flow models^10^. For example compared to CTA, CA has spatial resolution of 150-200 *mu*m vs 300-400 *mu*m and temporal resolution of 10ms vs 80-190ms^10^. Therefore, deriving 3D arterial geometries based on CA renders itself to the capability of reconstructing complex anatomic regions and including greater number of side branches^11^. Accurate anatomy has a direct impact on the fidelity of CFD simulations, thus by relying on CA data, we were able to simulate flow in complex coronary lesions. We achieve this by using a validated 3D reconstruction algorithm as described in^9^ that uses pair of 2D angiograms and creates the 3D coronary skeleton by detecting vessel centerlines and cross-sectional diameter. This reconstruction algorithm enables capturing anatomic details of third and fourth order branching patterns and vessel tortuousity up to 1mm in diameter. However, a closely related to simulating flow in these complex 3D arterial geometries is that it poses formidable computational load. To this end, we coupled our CA reconstructions with a massively parallel CFD solver that efficiently scales on millions of processors and can accurately simulate flow in different arterial vasculature^11,17,18,50^. HARVEY (v2) has previously been validated with *in vitro* experiments in patient-derived 3D vascular geometries^11,17,18^. Despite the complexity and anatomic detail of arterial models, these geometries could be used without the need for mesh refinement on exterior surfaces because HARVEY (v2) is nearly agnostic to mesh triangulation. Such features enable integration of flow from smaller side branches to the main branch flow, creating realistic arterial tree physiology. Therefore, we methodologically advance existing state-of-art studies, which non-invasively compute diagnostic metrics using angiograms for 3D arterial reconstruction.

Previous studies that have used routine angiographic data for non-invasive physiological assessment of coronary arteries typically use a simplified geometric model incorporating only the main vessel-of-interest with branching up to a single side vessel^43,45^. Such single vessel arterial models preclude the inclusion of collateral vessel bed and bifurcation lesions. Other angiogram-based studies, which incorporate an arterial tree up to first or second order vessel branching, have used a reduced-order lumped parameter model for computational modeling^6,51^. In reduced order models, the fluid dynamic equations are not solved, and instead the arterial network is treated as an electrical circuit and the vessel segments as resistors, which lowers the computational complexity^51,52^. With reduced order models, local intra-arterial physiology, WSS and velocity fields, cannot be obtained. Therefore, we note that existing CA-based computational studies either rely on a simplified 3D geometry or computational algorithm. Such simplifications potentially limit any complex lesion and local hemodynamic analysis for arterial networks and quantities, for example ESS and velocity, cannot be derived^52,53^. Therefore, to the best of our knowledge, this is the first CA-based 3D CFD method to simulate hyperemia and compute physiological flow parameters in complex and simple, single coronary lesions.

### Model robustness and feasibility for integration in clinical catheterization laboratory

We validate CFD-CA methodology by comparing the derived FFR and resting gradient values to invasively measured FFR and resting gradients. We report no difference in the accuracy of calculating resting pressure gradients (average error 3.1%, r2 correlation = 0.67, mean difference = 0.003, standard deviation = 0.07) and FFR (average error 6.7%, r2 correlation 0.59, mean difference = 0.02, standard deviation = 0.06) for both simple and complex lesion groups. These results hold equivalent-to-better limits of agreement with other current state-of-the-art computational FFR models^6,43–46,51^. We establish the robustness of our CFD-CA methodology by performing sensitivity analysis and report near independence of computed FFR with respect to variability in clinical measurements, such as cardiac output, hematocrit and heart rate ($<2\%$) on per-patient basis. Overall, using only readily available clinical record data and conventional angiogram data, this methodology also lacks the need for sophisticated diagnostic machinery.

### Clinical implications for hemodynamic evaluation in complex coronary lesions

By deriving the local hemodynamic physiology, we were able to study for the first time previously intractable relationship between complex and non-complex coronary lesions for pressure-based metrics, such as FFR and intra-arterial hemodynamics on a per-patient basis. This investigation is significant for two reasons:

- Traditional diagnostic metrics such as resting gradient and FFR do not significantly differ between complex and non-complex lesions. Therefore, we investigated intra-arterial hemodynamics differences, such as ESS and velocity, which can help understand the reason for worsened clinical outcome of patients with complex lesions such as bifurcation, ostial and serial lesions.
- The physiological information, in the form of pressure, ESS and velocity for the complete arterial tree in primary, secondary and tertiary branches, is not clinically available to physicians. This analysis can be utilized to study prospective progression of atherosclerosis in arterial segments secondary to the main diseased vessels.

### Study limitations and future directions

There are several limitation in this study. First, wall motion is not considered this study. However, rigid walls is a common assumption used in computational fluid dynamic studies evaluating coronary hemodynamics and FFR, as it is not highly sensitive to hemodynamic conditions such as heart rate, blood pressure, and myocardial contractility^25,26,34^. Second limitation is the small number of patient cases, and the high diagnostic accuracy reported for the 14 patient datasets may be serendipitous. Furthermore, the complex lesion also excluded vulnerable plaques because CA cannot easily detect calcification^54^. However, despite of excluding vulnerable plaques and the small cohort size, the patient population in this study represents a different complex lesion types and the boundary conditions were uniquely tuned to each patient case. Thus, the results reported here are encouraging and hypothesis-generating, and warrant study in a larger patient cohort with outcome data.

While effectiveness and robustness of the LBM for computing ESS in vascular regimes is well-established^55–57^, another limitation of this work is that our CFD-CA FFR validation using invasive measurements does not directly establish the accuracy of velocity and ESS. However, in our previous works we validated HARVEY simulation using 3D printing and experimental results for physiologically relevant flows in vascular geometries^18,50^. In these works, complete velocity profile was validated with particle image velocimetry (PIV) data using physiological blood flow profiles and 3D printed aorta and femoral geometries, which differ in the type of flow and physical scale. As for the ESS calculation, the strain rate tensor $S = (1/2)( \nabla \mathbf {v} + (\nabla \mathbf {v})^T )$ is used in HARVEY, therefore we believe these experimental comparisons supports the accuracy of both velocity and ESS using HARVEY. Other inherent limitation of the LBM-based CFD solvers is that the time step size is severely limited by the Courant–Friedrichs–Lewy number which results in large number of total time steps and thus longer timescales^58^. Therefore, the computational results are currently obtained offline, however, the use of massively parallel CFD solver enables near real-time ( 30 minutes) analysis.

The long-term goal of this study is to improve patient outcome by developing patient-specific multiscale CFD models based on conventional CA data. This approach is significant as the CFD-CA methodology enables seamless integration with routine cardiac catheterization laboratories by relying on conventional angiogram data. The proposed CFD technology solves fluid dynamic equations on every point of the 3D arterial geometry for patient cases displaying any degree of anatomic lesion complexity. Such unique features provide a complete road map of coronary physiology which describes the lesion-specific vessel and downstream effects on other vessels in the arterial tree. Our framework has minimal manual interaction for setting parameters and negligible variance with respect to uncertainties in model inputs. This invariance provides high prediction reliability of key hemodynamic risk factors and diagnostic metrics which is crucial for wide scale deployment in clinical decision-making.

## Methods

### Study population

This study does not involve any experiments on humans and/or the use of human tissue samples. This study was approved by the Partners Human Research Committee Institutional Review Board who waived informed consent for the study (IRB Protocol $\#$2015P001084). It was performed in accordance with relevant guidelines and regulations as per the approved IRB protocol. The study uses de-identified and anonymized coronary angiography imaging datasets from 14 patients who underwent both a clinically indicated coronary angiography and FFR procedure at Brigham and Women’s Hospital. The 14 patients were divided into two groups of 7 patients cases depending if the lesion was either simple and complex lesion. Complex lesion included: ostial lesion, bifurcation lesion, serial lesion, target lesion vessels supplied by collateral flow due to total occlusion or anatomy variant. Acquired patient angiograms included a minimum of 4 standard orthogonal views of the left coronary circulation and 2 standard orthogonal views of the right coronary circulation. FFR was performed using intravenous adenosine to induce maximal hyperemia.

The location of distal pressure *Pd* measurement was provided by an experienced interventional cardiologist. Particularly, in serial lesions, pressure was measured to the most distal lesion of the two or three lesions present in the vessel. However, in the case bifurcation lesions FFR could be measured in either the larger main vessel or in the marginal side branch^59,60^. The particular bifurcation lesion case included in this study involved the LAD vessel and the diagonal 1 branch, and FFR was measured in the LAD. Clinical variables, including blood pressure, heart rate, hematocrit, and FFR data at the time of coronary angiography and FFR measurement were collected.

### Model reconstruction and validation

For angiogram reconstructions, we applied a 3D reconstruction algorithm described in^9,15^. This 3D reconstruction algorithm is automated, and the segmentation is deterministic without the need of manual intervention. The method uses a pair of 2D angiograms and parameters of the C-arm gantry system (e.g., separation angles > 45$^{\circ }$, distance between X-ray spot and image intensifier, and pixel size) to create the 3D coronary skeleton by detecting vessel centerlines and cross-sectional diameter. Reconstructions were obtained at end-diastole. All geometric models were exported as mesh files in standard stereolithography file format for further geometric and CFD analysis as shown in supplementary figure 1 and 2. The topological and anatomical validity of the angiogram models compared to the corresponding angiograms was assessed by calculating the minimal luminal diameter and reference diameter derived from the 3D models, and the biplane angiogram data were aligned using ImageJ (v1.52k).

### Computational fluid dynamics solver

CFD simulations were performed using HARVEY (v2), a computational hemodynamics software package^12,13,17^. HARVEY (v2) (v2) is based on the LBM, an alternate approach to solving the Navier-Stokes equations governing the fluid flow. Derived from the Boltzmann equation of kinetic theory, LBM describes the fluid by a probability distribution function $f_i$ of particles moving on a regular lattice *x* according to discrete velocities $c_i$. Macroscopic fluid variables, such as the pressure *p* and velocity *u*, are computed locally as moments of $f_i(\mathbf {x},t)$. The evolution of the distribution $f_i(\mathbf {x},t)$ over time $\Delta t$ is governed by the lattice Boltzmann equation^61^:

1 $$\begin{aligned} f_{i}(\mathbf {x}+\mathbf {c}_i \Delta t, t + \Delta t) - f_i(\mathbf {x},t) = -\Omega (f_i(\mathbf {x},t)-f_{i}^{eq}(\mathbf {x},t)) \end{aligned}$$

for the collision operator and Maxwell-Boltzmann equilibrium distribution $f_{i}^{eq}(\mathbf {x},t)$. A comprehensive explanation of the LBM may be found in^61^. The HARVEY (v2) implementation uses the standard three dimensional D3Q19 velocity discretization and the single relaxation time BGK collision kernel. The code is written in C and C++, using OpenMP and MPI for parallelization. Wall shear stress is evaluated at fluid points adjacent to the wall. The wall shear stress vector $\mathbf {\tau _i}$ is computed on-site in our LBM solver, using the equation^57^ :

2 $$\begin{aligned} \tau _i = -\frac{\mu \omega }{c_s^2 \rho } f_{\alpha }^{neq} c_{\alpha j} n_j (c_{\alpha i} - c_{\alpha k} n_i n_k) \end{aligned}$$

with non-equilibrium distribution function $\mathbf {f}^{neq}$, dynamic viscosity $\mathbf {\mu }$ and fluid density $\mathbf {\rho }$, BGK relaxation rate $\omega$, lattice speed of sound $c_s$, components $c_{\alpha i}$ of the discrete velocity vector $\mathbf {c}_i$, and the outward normal vector $\mathbf {n}$^57^. The pressure in lattice units was converted into physical units (mmHg) by using the pressure coefficient equation:

3 $$\begin{aligned} C_p =\frac{\Delta P}{\frac{1}{2} \rho u^{2}} \end{aligned}$$

where $\Delta P$ is the pressure difference, and $\rho$ and *u* are the reference density and velocity, respectively. Further details about the numerical implementation, parallelization, and scaling of HARVEY (v2) can be found in^12,13,17^.

### Model assumptions

To model the coronary circulation, we simulated blood as an incompressible Newtonian fluid with density of 1060 kg/m$^3$. The blood vessels were modeled as rigid walls with a no-slip boundary condition at lateral surfaces. These are valid assumptions often made in CFD studies due to the shear rates found in vessels of the size of the coronaries^25,26^.

### Personalized hemodynamic simulations

It is important to set up a CFD simulation individualized for each patient case by incorporating: cardiac output, heart rate, coronary dominance, blood pressure and vessel sizes. For the inlet boundary condition, the transient flow was considered using velocity waveform tuned to the patient parameters. Transient (or pulsatile) simulations are more realistic than steady simulations as they capture the periodically changing velocity during the cardiac cycle. Pulsatile ESS and pressure derived from transient simulations were thus varying in magnitude, unidirectional and averaged over the period of the cardiac cycle. A Poiseuille profile was prescribed at the inlet and finite difference boundary conditions were imposed^28,62^. The Poiseuille equation is given as^28^:

4 $$\begin{aligned} R = \frac{8 \eta \ell }{\pi r^4 } \end{aligned}$$

where, *R* is resistance of blood with viscosity $\eta$ flowing through vessel of length, $\ell$ and is inversely proportional to the fourth power of radius^28^. Vessel radius has a stronger effect on the overall flow compared to vessel length and viscosity, such that small changes in radius result in dramatic changes in vessel resistance and overall volume. This equation provides an important description for relating flow with vessel anatomy. With the assumption of Newtonian flow, it can be used for modeling physiological (e.g., vascular constriction) and pathological (e.g., arterial stenosis) changes based on vessel radius and how that affect pressure and flow volume^28^.

At the outlets, a lumped parameter model was prescribed using microcirculation resistance^25,28–30,63^. The resistance based boundary conditions assume linear relationship between flow rate and pressure, but can be uniquely prescribed to each vessel outlet of the arterial network. Such an implementation for coronary circulation, while computationally more expensive than constant pressure boundary conditions, is needed to ensure the physiological behavior of arterial network as each arterial outlet offers different flow resistance and ESS due to different radii of arterial outlets^63^. To prevent noise, we averaged the diameter for each arterial outlet up to a length of 1.5mm from the vessel endings. Thus, for all patient cases, the coronary arterial microcirculation resistance *(R)* for each outlet *i* was computed based on the vessel diameter, mean flow *(Q)* and mean aortic pressure *(P)* using the following equation^25,63^:

5 $$\begin{aligned} P_i=Q_i.R_i \end{aligned}$$

To numerically determine grid-invariant solutions is important to perform accurate computational simulations. Therefore, a convergence study was performed to obtain grid-invariant solutions, the results of which indicate that simulations were convergent at 50 $\mu$m resolution, with approximately $1.9\times 10^7$ and $1.6\times 10^7$ fluid points for LCA and RCA models, respectively. To this end, temporal and spatial convergence tests were performed, the results of which are shown in supplementary figure 3a,b. As flow in complex vasculature does not have an analytical solution, we used a high-resolution grid as the reference solution for spatial convergence and a long simulation for temporal convergence. These references for a coronary geometry corresponded to a physical resolution of 40 *mu*m as the reference for spatial convergence and 5 cardiac cycles ($10^5$ time steps) for temporal convergence. A distal location in the arterial vessel was selected for measuring convergence to ensure flow was fully developed along the arterial ends. Temporal convergence was noted at $20\times 10^3$ (second cardiac cycle) time steps and spatial convergence at 50 $\mu$m with L2 error $<10^{-3}$. Further, numerical tests related to convergence and stability in a coronary artery using HARVEY have been demonstrated in our previous work^18,50^. Due to the large fluid domain, simulations were conducted on 1024 cores of Intel Xeon E5-2699 processors with 56 Gb/s Infiniband interconnect on the Duke Compute Cluster.

### Statistical analysis

Kolmogorov-Smirnov analysis was used to test for the normal distribution. Correlation between invasively measured FFR and predicted FFR was determined using linear regression. Deming regression was used to assess difference between invasive and CFD-CA FFR. To demonstrate absolute agreement between invasive and CFD-CA FFR, Bland Altman analysis was used and the 95% confidence intervals of agreements were calculated. Estimated bias was calculated as the mean difference between the invasive FFR (independent variable) and CFD-CA FFR (dependent variable). Continuous variables, such as the ESS and velocity, were described by mean and standard deviation. A two-tailed paired ratio t-test was used to assess whether mean differences between local hemodynamic quantities (ESS and velocity) were significant. A value of $p<$0.05 is considered significant.

## Supplementary Information

Supplementary Information

## References

[1] Louvard Y, Medina A (2015). Definitions and classifications of bifurcation lesions and treatment. EuroInterv. J. EuroPCR Ccllab. Work. Group Interven. Cardiol. Eur. Soc. Cardiol..

[2] Iftikhar, S. F. & Hu, P. Complex coronary artery lesions. (2019). 30969721

[3] Katritsis DG (2012). Flow patterns at stented coronary bifurcations: computational fluid dynamics analysis. Circul. Cardiovasc. Interv..

[4] Dash D (2014). Recent perspective on coronary artery bifurcation interventions. Heart Asia.

[5] Coenen A (2018). Diagnostic accuracy of a machine-learning approach to coronary computed tomographic angiography–based fractional flow reserve: result from the machine consortium. Circu. Cardiovasc. Imag..

[6] Fearon WF (2019). Accuracy of fractional flow reserve derived from coronary angiography. Circulation.

[7] Dishmon DA, Elhaddi A, Packard K, Gupta V, Fischell TA (2011). High incidence of inaccurate stent placement in the treatment ofcoronary aorto-ostial disease. J. Invas. Cardiol..

[8] Wong P (2008). Two years experience of a simple technique of precise ostial coronary stenting. Catheter. Cardiovasc. Interv.

[9] Chen SJ, Carroll JD (2000). 3-d reconstruction of coronary arterial tree to optimize angiographic visualization. IEEE Trans. Med. Imag..

[10] Stefanini GG, Windecker S (2015). Can coronary computed tomography angiography replace invasive angiography?: Coronary computed tomography angiography cannot replace invasive angiography. Circulation.

[11] Vardhan M (2019). The importance of side branches in modeling 3d hemodynamics from angiograms for patients with coronary artery disease. Sci. Rep..

[12] Randles, A. P., Kale, V., Hammond, J., Gropp, W. & Kaxiras, E. Performance analysis of the lattice boltzmann model beyond navier-stokes. In *Parallel & Distributed Processing (IPDPS), 2013 IEEE 27th International Symposium on*, 1063–1074 (IEEE, 2013).

[13] Gounley J (2019). Computing the ankle-brachial index with parallel computational fluid dynamics. J. Biomech..

[14] Carroll, J. & Chen, S.-Y. J. Method and apparatus for three-dimensional reconstruction of coronary vessels from angiographic images and analytical techniques applied thereto (2002). US Patent 6,501,848.

[15] Green NE (2005). Angiographic views used for percutaneous coronary interventions: a three-dimensional analysis of physician-determined vs. computer-generated views. Catheter. Cardiovasc. Interv..

[16] Schneider CA, Rasband WS, Eliceiri KW (2012). Nih image to imagej: 25 years of image analysis. Nat. Methods.

[17] Randles, A., Draeger, E. W., Oppelstrup, T., Krauss, L. & Gunnels, J. A. Massively parallel models of the human circulatory system. In *Proc. International Conference for High Performance Computing, Networking, Storage and Analysis*, 1 (ACM, 2015).

[18] Gounley, J. *et al.* Does the degree of coarctation of the aorta influence wall shear stress focal heterogeneity? In *Engineering in Medicine and Biology Society (EMBC), 2016 IEEE 38th Annual International Conference of the*, 3429–3432 (IEEE, 2016). 10.1109/EMBC.2016.7591465 PMC5905411 28269039

[19] Gounley, J., Vardhan, M. & Randles, A. A computational framework to assess the influence of changes in vascular geometry on blood flow. In *Proceedings of the Platform for Advanced Scientific Computing Conference*, 2 (ACM, 2017).

[20] Gounley, J., Vardhan, M. & Randles, A. A framework for comparing vascular hemodynamics at different points in time. *Computer Physics Communications* (2018). 10.1016/j.cpc.2018.05.014 PMC6261380 30504967

[21] Donath S (2009). walberla: the need for large-scale super computers. High Perf. Comput. Sci. Eng..

[22] Mazzeo MD, Coveney PV (2008). Hemelb: a high performance parallel lattice-Boltzmann code for large scale fluid flow in complex geometries. Comput. Phys. Commun..

[23] Melchionna S (2013). Risk assessment of atherosclerotic plaques based on global biomechanics. Med. Eng. Phys..

[24] Giannopoulos AA (2018). Diagnostic performance of a lattice boltzmann-based method for ct-based fractional flow reserve.. EuroIntervention J. EuroPCR Collab. Work. Group Interv. Cardiol. Eur. Soc. Cardiol..

[25] Taylor CA, Fonte TA, Min JK (2013). Computational fluid dynamics applied to cardiac computed tomography for noninvasive quantification of fractional flow reserve: scientific basis. J. Am. College Cardiol..

[26] Eslami, P. *et al.* Effect of wall elasticity on hemodynamics and wall shear stress in patient-specific simulations in the coronary arteries. *J. Biomech. Eng.* (2019). 10.1115/1.4043722 PMC7105147 31074768

[27] Razavi M, Shirani E, Kassab GS (2018). Scaling laws of flow rate, vessel blood volume, lengths, and transit times with number of capillaries. Front. Physiol..

[28] Klabunde, R. *Cardiovascular Physiology Concepts* (Lippincott Williams & Wilkins, 2011).

[29] Kamiya A, Togawa T (1980). Adaptive regulation of wall shear stress to flow change in the canine carotid artery. Am. J. Physiol.-Heart Circul. Physiol..

[30] Kim HJ (2009). On coupling a lumped parameter heart model and a three-dimensional finite element aorta model. Ann. Biomed. Eng..

[31] Wilson RF, Wyche K, Christensen BV, Zimmer S, Laxson DD (1990). Effects of adenosine on human coronary arterial circulation. Circulation.

[32] Frauenfelder T (2007). In-vivo flow simulation in coronary arteries based on computed tomography datasets: feasibility and initial results. Eur. Radiol..

[33] Pijls NH (2007). Percutaneous coronary intervention of functionally nonsignificant stenosis: 5-year follow-up of the defer study. J. Am. College Cardiol..

[34] de Bruyne B (1996). Simultaneous coronary pressure and flow velocity measurements in humans: feasibility, reproducibility, and hemodynamic dependence of coronary flow velocity reserve, hyperemic flow versus pressure slope index, and fractional flow reserve. Circulation.

[35] Malek AM, Alper SL, Izumo S (1999). Hemodynamic shear stress and its role in atherosclerosis. Jama.

[36] Chatzizisis YS (2007). Role of endothelial shear stress in the natural history of coronary atherosclerosis and vascular remodeling: molecular, cellular, and vascular behavior. J. Am. College Cardiol..

[37] Wentzel JJ (2012). Endothelial shear stress in the evolution of coronary atherosclerotic plaque and vascular remodelling: current understanding and remaining questions. Cardiovasc. Res..

[38] Van de Hoef TP, Siebes M, Spaan JA, Piek JJ (2015). Fundamentals in clinical coronary physiology: why coronary flow is more important than coronary pressure. Eur. Heart J..

[39] Koskinas KC (2009). The role of low endothelial shear stress in the conversion of atherosclerotic lesions from stable to unstable plaque. Curr. Opin. Cardiol..

[40] Lafont, A. & Topol, E. J. *Arterial Remodeling: A Critical Factor in Restenosis* Vol. 198 (Springer Science & Business Media, 2012).

[41] Kumar A (2018). High coronary shear stress in patients with coronary artery disease predicts myocardial infarction. J. Am. College Cardiol..

[42] Kang D-Y (2018). Impact of coronary lesion geometry on fractional flow reserve: data from interventional cardiology research in-cooperation society-fractional flow reserve and intravascular ultrasound registry. Circu. Cardiovasc. Imag..

[43] Tröbs M (2016). Comparison of fractional flow reserve based on computational fluid dynamics modeling using coronary angiographic vessel morphology versus invasively measured fractional flow reserve. Am. J. Cardiol..

[44] Morris PD (2013). Virtual fractional flow reserve from coronary angiography: modeling the significance of coronary lesions: results from the virtu-1 (virtual fractional flow reserve from coronary angiography) study. JACC Cardiovasc. Interv..

[45] Papafaklis MI (2014). Fast virtual functional assessment of intermediate coronary lesions using routine angiographic data and blood flow simulation in humans: comparison with pressure wire-fractional flow reserve. EuroIntervention J. EuroPCR Collab. Work. Group Interv. Cardiolo. Eur. Soc. Cardiol..

[46] Tu S (2016). Diagnostic accuracy of fast computational approaches to derive fractional flow reserve from diagnostic coronary angiography: the international multicenter favor pilot study.. JACC Cardiovasc. Interv..

[47] Han, D. *et al.* Relationship between endothelial wall shear stress and high-risk atherosclerotic plaque characteristics for identification of coronary lesions that cause ischemia: A direct comparison with fractional flow reserve. *J. Ame. Heart Assoc.* **5**, (2016). 10.1161/JAHA.116.004186 PMC5210401 27993831

[48] Gijsen F (2019). Expert recommendations on the assessment of wall shear stress in human coronary arteries: existing methodologies, technical considerations, and clinical applications. Eur. Heart J..

[49] Lee JM (2019). Identification of high-risk plaques destined to cause acute coronary syndrome using coronary computed tomographic angiography and computational fluid dynamics. JACC Cardiovasc. Imag..

[50] Feiger, B. *et al.* Suitability of lattice boltzmann inlet and outlet boundary conditions for simulating flow in patient-specific vasculature. *Int. J. Numer. Methods Biomed. Eng.* e3198 (2019). 10.1002/cnm.3198 PMC7605305 30838793

[51] Pellicano M (2017). Validation study of image-based fractional flow reserve during coronary angiography. Circu. Cardiovasc. Interv..

[52] Kokalari I, Karaja T, Guerrisi M (2013). Review on lumped parameter method for modeling the blood flow in systemic arteries. J. Biomed. Sci. Eng..

[53] Xiao N, Alastruey J, Alberto Figueroa C (2014). A systematic comparison between 1-d and 3-d hemodynamics in compliant arterial models. Int. J. Numer. Methods Biomed. Eng..

[54] Budoff MJ (2008). Diagnostic performance of 64-multidetector row coronary computed tomographic angiography for evaluation of coronary artery stenosis in individuals without known coronary artery disease: results from the prospective multicenter accuracy (assessment by coronary computed tomographic angiography of individuals undergoing invasive coronary angiography) trial. J. Am. Coll. Cardiol..

[55] Boyd J, Buick J, Cosgrove J, Stansell P (2005). Application of the lattice boltzmann model to simulated stenosis growth in a two-dimensional carotid artery. Phys. Med. Biol..

[56] Stahl B, Chopard B, Latt J (2010). Measurements of wall shear stress with the lattice boltzmann method and staircase approximation of boundaries. Comput. Fluids.

[57] Matyka M, Koza Z, Mirosław Ł (2013). Wall orientation and shear stress in the lattice boltzmann model. Comput. Fluids.

[58] Fakhari A, Lee T (2015). Numerics of the lattice boltzmann method on nonuniform grids: standard lbm and finite-difference lbm. Comput. Fluids.

[59] Park SH, Koo B-K (2012). Clinical applications of fractional flow reserve in bifurcation lesions. J. Geriatric Cardiol. JGC.

[60] Achenbach S (2017). Performing and interpreting fractional flow reserve measurements in clinical practice: an expert consensus document. Interv. Cardiol. Rev..

[61] Chen S, Doolen GD (1998). Lattice boltzmann method for fluid flows. Ann. Rev. Fluid Mech..

[62] Latt, J., Chopard, B., Malaspinas, O., Deville, M. & Michler, A. Straight velocity boundaries in the lattice boltzmann method. *Phys. Rev. E* **77**, (2008). 10.1103/PhysRevE.77.056703 18643191

[63] Grinberg L, Karniadakis GE (2008). Outflow boundary conditions for arterial networks with multiple outlets. Ann. Biomed. Eng..

