# Supplementary material for: Non-invasive characterization of complex coronary lesions
Source: Sci Rep. 2021 Apr 14;11:8145. doi: 10.1038/s41598-021-86360-6 (PMC8047040; doi:10.1038/s41598-021-86360-6)
Supplement: Supplementary file 1 — Supplementary Information [file 41598_2021_86360_MOESM1_ESM.pdf]

-- Full title –

Non-invasive characterization of complex coronary lesions

-- First author's surname and short title:

Vardhan, “Non-invasive characterization of complex coronary lesions”

-- Authors' names, academic degrees, and affiliations

Madhurima Vardhan, MS, Department of Biomedical Engineering, Duke University
John Gounley, PhD, Computational Sciences and Engineering Division, Oak Ridge National Laboratory
S. James Chen, PhD, Department of Medicine/Cardiology, University of Colorado, Denver
Andrew M. Kahn, MD, Division of Cardiovascular Medicine, University of California San Diego
Eric C. Chi, PhD, Department of Statistics, North Carolina State University, 27695, USA
Jane A. Leopold, MD, Division of Cardiovascular Medicine, Brigham and Women’s Hospital, Harvard Medical School
Amanda Randles, PhD, Department of Biomedical Engineering, Duke University

-- Name and complete address for correspondence (include street name and address as well as post office box, and address for reprints if different from correspondence)

Amanda Randles,
303 Gross Hall, 140 Science Dr., Durham, NC 27708

-- Fax number, telephone number and email address

(919) 660-5425 amanda.randles@duke.edu

-- Disclosure statement

Authors report no conflicts.

Supplementary Table 1: Patient Parameters

Case	Vessel	Hematocrit (percent)	Cardiac output (L/min)	Heartrate (resting in bpm)	Heartrate (hyperemia in bpm)	Blood pressure (resting in mmHg)	Blood pressure (hyperemia in mmHg)	Ostium diameter (mm)	Coronary dominance	Lesion type
Case 1	LAD	42.2	4.04	70	101	133/72	113/65	4.17	Right	Single LAD lesion
Case 2	RCA	38	4.1	82	93	134/83	90/63	4.16	Right	Single RCA lesion
Case 3	LCx	37	3.15	85	87	111/57	101/62	4.16	Co-dominant	Serial lesion LCx
Case 4	RCA	37	3.15	85	87	111/57	90/50	3.47	Co-dominant	Ostial lesion RCA
Case 5	LAD	42.4	3.75	119	113	128/65	132/72	5.31	Left	Single LAD lesion
Case 6	RCA	42.5	3.58	56	54	95/48	73/38	3.37	Right	Single RCA lesion
Case 7	Left Main	34.2	3.12	64	106	130/83	155/80	4.93	Right	Left main, serial lesion in LAD
Case 8	LCx	33.3	3.9	57	60	129/65	113/63	3.17	Right	Collateral flow, single LCx lesion
Case 9	RCA	38.09	2.62	79	80	122/64	120/68	2.21	Right	Single RCA lesion
Case 10	LAD	36.1	5.35	48	55	128/59	113/56	5.02	Right	Serial LAD lesion
Case 11	LAD	29.6	4.62	84	87	160/71	118/56	5.61	Left	Bifurcation lesion and serial lesion LAD
Case 12	RCA	37.9	6.29	61	67	191/86	137/74	4.47	Right	Triple serial lesion RCA
Case 13	LAD	41.2	5.28	72	75	141/69	101/48	4.42	Right	Serial lesion LAD
Case 14	RCA	40.1	2.54	54	94	128/63	145/82	4.06	Right	Single RCA lesion

Supplementary Table 2: Percent Stenoses

Case	Vessel	Patient angiogram	Reconstructed STL mesh
Case 1	LAD	12%	14%
Case 2	RCA	51%	52%
Case 3	LCx	94%, 53%	95%, 55%
Case 4	RCA	61%	60%
Case 5	LAD	43%	37%
Case 6	RCA	19%	19%
Case 7	Left Main	70%, 60%	72%, 65%
Case 8	LCx	22%	25%
Case 9	RCA	67%	60%
Case 10	LAD	70%, 50%	72%, 51%
Case 11	LAD	52%, 62%, 58%	50%, 64%, 60%
Case 12	RCA	43%, 61%, 59%	45%, 60%, 62%
Case 13	LAD	32%, 41%	30%, 43%
Case 14	RCA	60%	62%

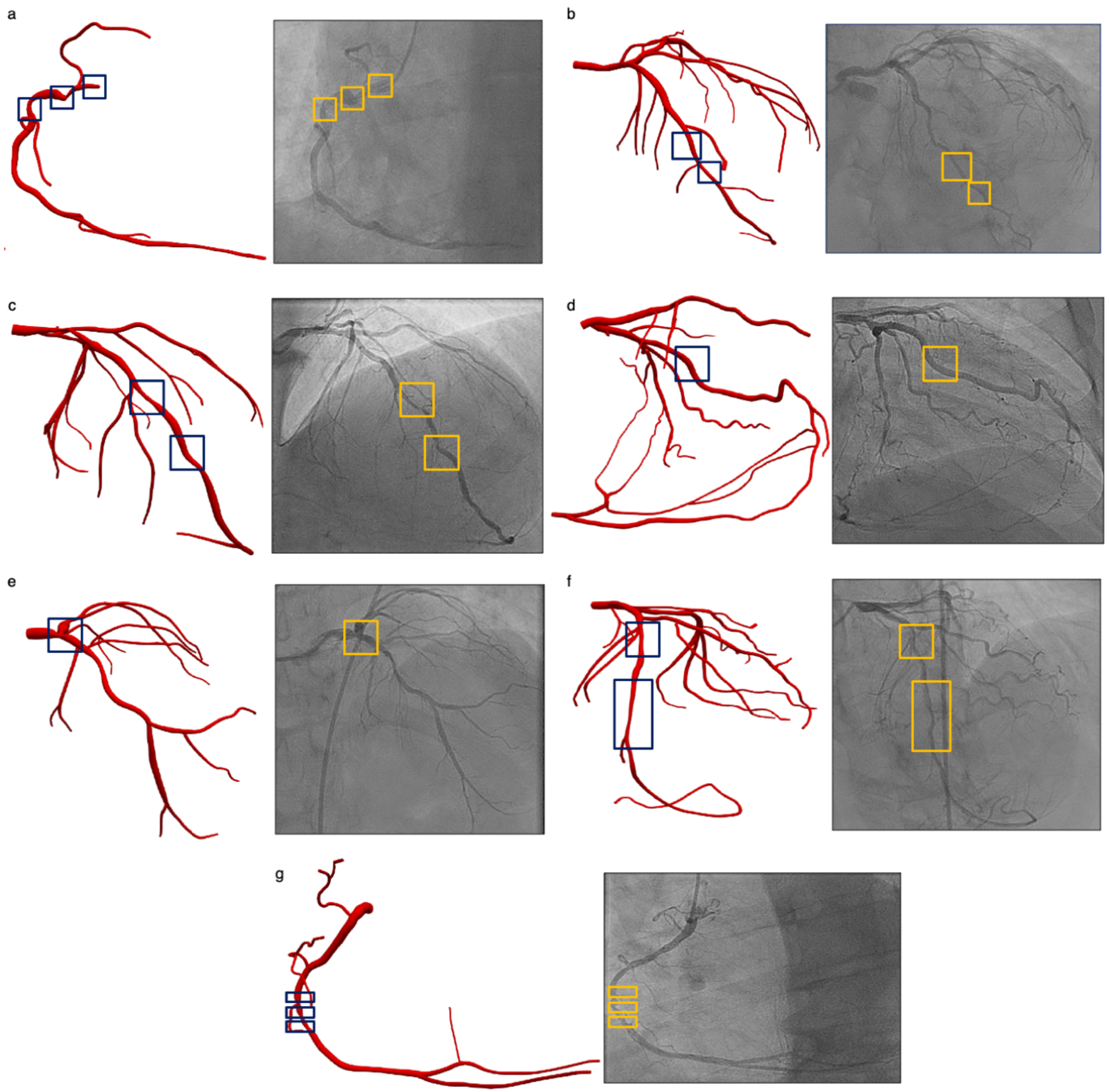

Supplementary figure 1: Complex coronary lesions with boxes identifying the location of lesion on the 3D geometry (left) and patient angiogram (right). a) Ostial and proximal serial lesions b) serial lesions in the LAD c) serial lesions in the LAD d) Lesion where blood supplied to RCA vascular bed via collaterals e) Bifurcation lesion involving Diagonal 1 branch f) serial lesions in the LAD g) serial lesions in the RCA.

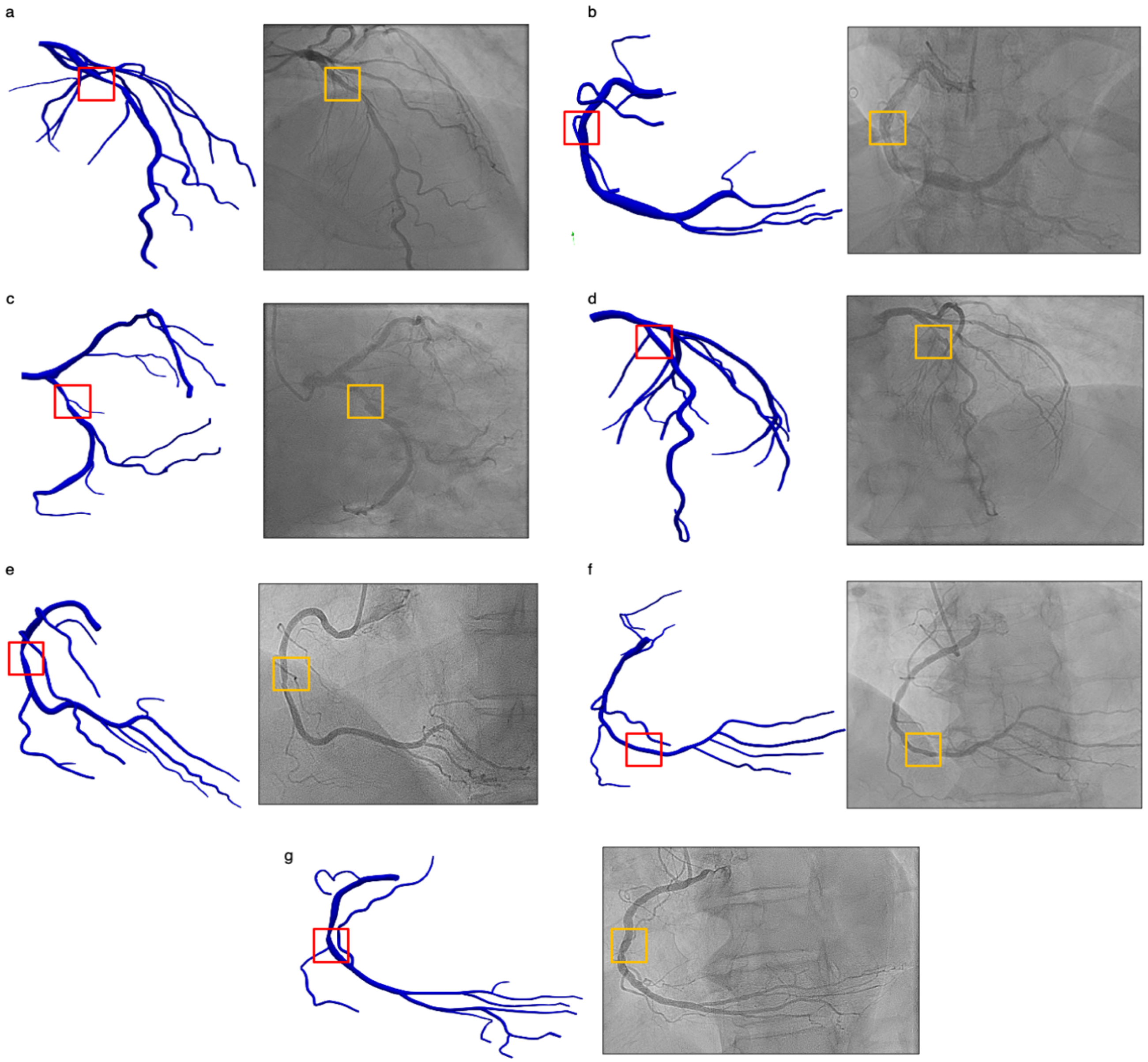

Supplementary figure 2: Simple, single coronary lesions with boxes identifying the location of lesion on the 3D geometry (left) and patient angiogram (right). a) LAD lesion b) RCA lesion c) LCx lesion d) LAD lesion e) RCA lesion f) RCA lesion g) RCA lesion.

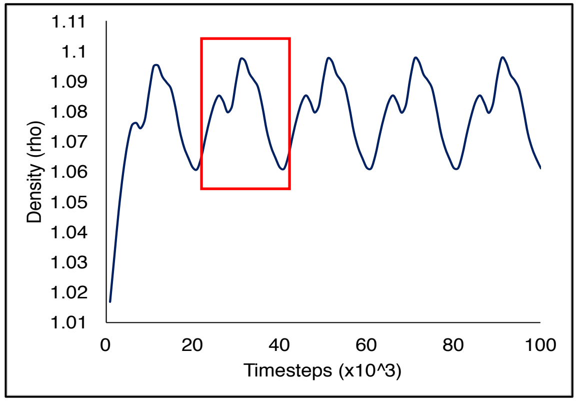

Grid spacing (in µm)	FFR (computed)	L_2 Error
100	0.974275526	0.007
80	0.982609052	0.003
70	0.979318638	0.002
50	0.979774628	0.001

(a) (b)

Supplementary figure 3: Numerical convergence. a) Temporal Convergence (pulsatile) for lattice Boltzmann density (rho). Density was used for computing fractional flow reserve (FFR) using the equation $C_p = \frac{\Delta P}{\frac{1}{2}\rho u^2}$, where ΔP is the pressure difference, and ρ and u are the reference density and velocity, respectively. Temporal convergence was achieved after the first pulse ($20\text{x}10^3$ timesteps), and the second pulse (identified by the red box) was used for computing FFR. b) Spatial convergence was noted at 50 µm for computing FFR with L_2 error $<10^{-3}$.

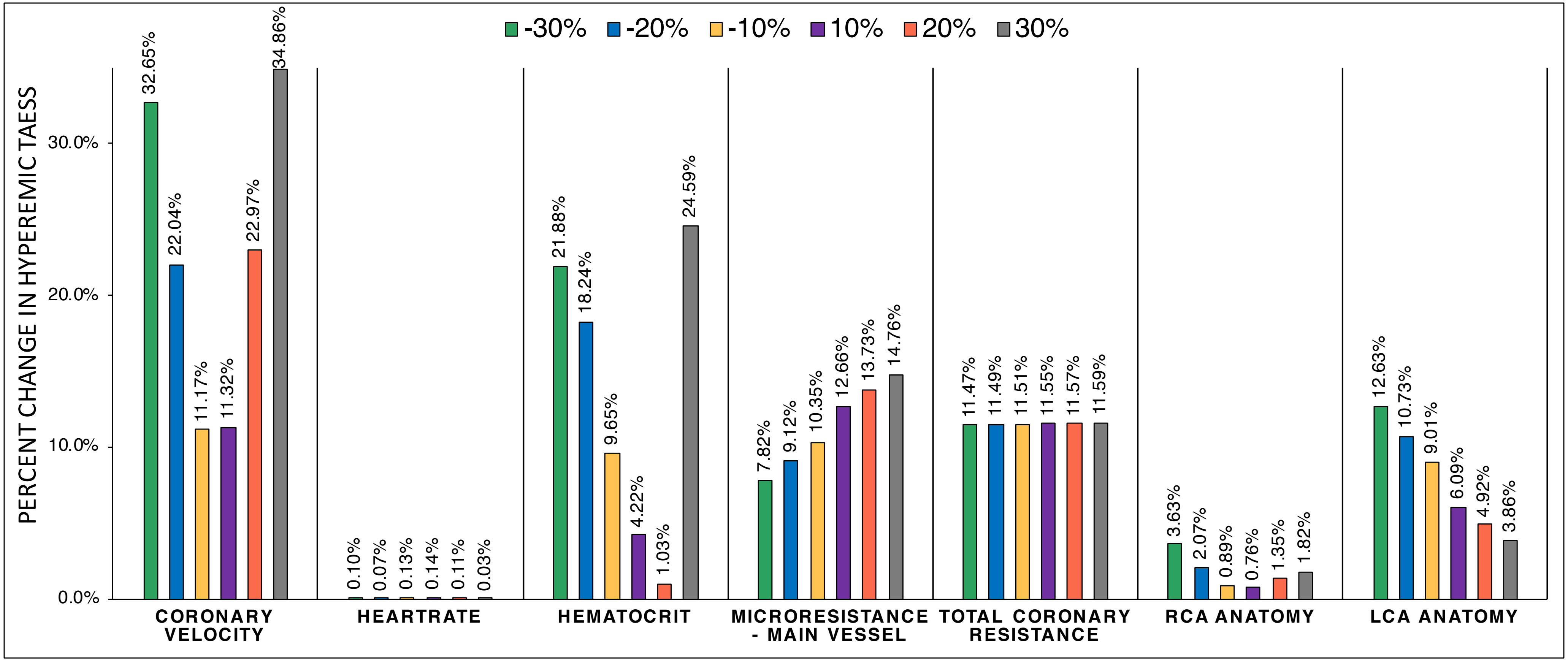

Supplementary figure 4: Sensitivity analysis for hyperemic time averaged endothelial shear stress (TAESS). Coronary anatomy is the driving feature for FFR predictions. Key hemodynamic input parameters: coronary velocity, heartrate, hematocrit and resistance were varied by ±10%, ±20%, and ±30%. To account for geometric influence percent stenosis was varied by ±10%, ±20%, and ±30%.

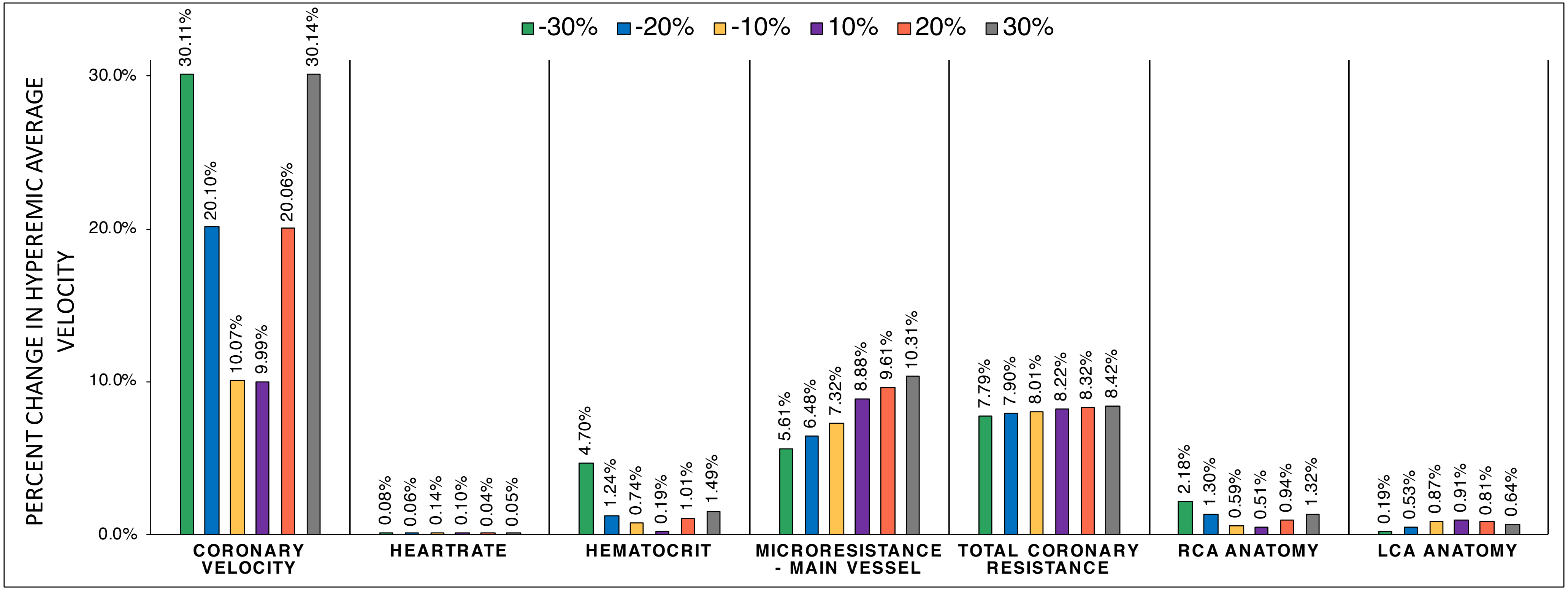

Supplementary figure 5: Sensitivity analysis for hyperemic average velocity. Coronary anatomy is the driving feature for FFR predictions. Key hemodynamic input parameters: coronary velocity, heartrate, hematocrit and resistance were varied by ±10%, ±20%, and ±30%. To account for geometric influence percent stenosis was varied by ±10%, ±20%, and ±30%.
